# A Shift From Logistic Software to Service Model: A Case Study of New Service-Driven-Software for Management of Emergency Supplies During Disasters and Emergency Conditions by WHO

**DOI:** 10.3389/fphar.2019.00473

**Published:** 2019-05-07

**Authors:** Huma Rasheed, Muhammad Usman, Waqas Ahmed, Muhammad Haroon Bacha, Aliya Zafar, Khalid Saeed Bukhari

**Affiliations:** ^1^Institute of Pharmaceutical Sciences, University of Veterinary and Animal Sciences, Lahore, Pakistan; ^2^United States Pharmacopeial Convention, Rockville, MD, United States; ^3^World Health Organization, Islamabad, Pakistan

**Keywords:** disaster and emergency management, logistics, inventory software, pharmaceutical supplies, supply chain management, essential medicines access

## Abstract

World Health Organization (WHO) states access to medicine as a priority area for universal health coverage, wherein a well-functioning medicine supply chain is indispensable. Optimization of supply chains to cut losses related to overstocking, expiration, and inefficiencies protect the investments and strengthen health systems to better deliver the health services. This article shares the experience of developing a service-driven-software for pharmaceutical supplies during emergency conditions and disasters, and the advantages gained. In 2005, Logistic Support System (LSS), the updated version of SUMA (Supply Management), was introduced by WHO during the earthquake in Pakistan which had offered valuable but limited services to many countries. Moving from *ad hoc* to a more organized approach, the medical donations and stockpiles of essential medicinal supplies were inventoried on LSS database for managing the dispatch of medical supplies to the disaster-hit area in a shortest possible time. Post disaster rescue and rehabilitation work further instigated the need for development of a new software, Pharmaceutical Information Management System (PIMS), that was effective in the emergency as well as routine inventory operations. It was used for efficient and improved access of medicines and faster decision making. The new systems proved vital to anticipate over/under stocking through proactive alerts and prompting. The updated information on epidemiological and drug utilization needs were crucial for the effective quantification and ordering throughout the supply chain. Implementation of PIMS demanded appreciable customization including conversion of system from stand-alone to online system with consolidation of information on stocks from all locations. Provision of multi-user option allowed facilitation according to the user authorization, and was equipped with improved-speed, efficiency, and security. PIMS was successfully replicated by the pioneer team of pharmacist from Pakistan in other countries.

## Introduction

Disaster relief program is required for the provision of medicine, food, tents, equipment, and other necessities of life to the victims in a misfortune area affected with earthquakes, floods, volcanic eruptions, wild fires, or other natural disasters. Disasters can also occur due to political and economic crisis, due to wars, political insurrection, or due to failure of government involving displacement and life-threatening situations for a large population. These natural and political disasters can affect any part of the world (Clay Whybark, 2007). Humanitarian logistic or supply chain management in disasters is the process of planning and implementation for the effective flow of costs and goods, dissemination of information from the point of origin to the point of consumption in order to meet the needs of victims, as well as the efficient storage and handling of materials and equipments. Logistics is the most expensive part of a disaster relief program and it covers almost 80% of the disaster relief cost (D’Uffizi et al., 2015). Efficiency of the flow of information between the supply chain units can be improved by the use of supply chain information systems (Sangiamkul and Van Hillegersberg, 2011). Pharmaceuticals and medical supplies are specialized category of goods requiring additional controls like product authenticity, registration status, expiry and batch records, storage specifications, adequacy of labeling in local language, controlled used and handling according to therapeutic categories in order to ensure the access of quality pharmaceuticals in appropriate number and suitable form at the point of care.

A disaster is a sudden, calamitous event that seriously disrupts the functioning of a community or society and causes human, material, and economic or environmental losses that exceed the community’s or society’s ability to cope using its own resources. Though often caused by nature, disasters can have human origins (International Federation of Red Cross and Red Crescent Societies, 2019). The magnitude of disaster is described in terms of people affected directly or indirectly. According to IFRCRCS a disaster is an event that devastates local resources, equal to or more than 10 people reported dead, more than 100 people reported affected, requesting assistance from national and international agencies and/or declaration of emergency. The unforeseen demand in terms of location, time, magnitude and type of disaster, the lack of resources (money, personnel, supplies, technology and transport), the risk associated with the immediate supplies and the search for wide variety of sources in short period of time are the main challenges in supply chain management during humanitarian crisis (Balcik and Beamon, 2008).

Coordination is a key factor that affects the success of humanitarian relief operation. Two types of coordination exist that are vertical and horizontal. In vertical type an organization coordinates in the form of chain at different levels while in horizontal coordination one organization coordinates with another in terms of resources, information, and services (Balcik et al., 2010).

Proficient management of inventory is essential to ensure safe and uninterrupted supply of medicine to the patients and healthcare providers during relief operations. Inventory management in the humanitarian relief operations, is challenged by unpredictability of demand, need of zero lead time (urgency of need), high variation in demand location, its quantity and the applicable lead times based on nature of stocks (Tayfur and Benjamin, 2007). Additional challenges are observed in humanitarian crisis supply chain management compared to the commercial or routine inventory management of pharmaceuticals. The non-availability of sufficient and suitable products can potentially impact the patient care leading to serious consequences even patient’s death in extreme cases. Holding of surplus stock, on the other hand, can also lead to expired products and wastage of resources (Fontaine et al., 2009; Stanger et al., 2012; Williamson and Devine, 2013; Ishii et al., 2017). Being specialized products the cost of disposal of expired and damaged pharmaceuticals is seen as a huge financial burden, sometimes more than the cost of the stocks involved (Pharmaciens Sans Frontières Comité International, 2005). Therefore, efficient inventory management is essential to reduce excessive cost and increase the effectiveness of the relief and rehabilitation programs.

The use of information technology is an essential component of managing pharmaceutical supplies in disaster relief programs these days. Sharing the computerized inventory information has substantially improved the efficiency and quality of error-prone processes (Chapman, 2007; Shih et al., 2009). Many tools, computer and manuals have been used for the coordination needed during the relief operations and aid programs. The specific tools designed for management of humanitarian supply chain include SUMA, LSS, LOGISTIX, Helios, UniTrack, and Sahana (Alexander, 2010).

Essential management team at World Health Organization (WHO), Islamabad identified several shortcomings of Logistic Support System (LSS) program during its use in post 2005 earth quake disaster relief operation in Pakistan and provided a trigger to the transformation of system to its advanced form named as Pharmaceutical Information Management System (PIMS). This new version integrates data collection, data processing, and presentation of information to make fast, and reliable, evidence-based decisions for the management of pharmaceutical services during disaster and emergency services at large scale. The PMIS ensures accountability by creating the audit trial of the products entering and leaving the pharmaceutical supply system (Bate et al., 2013) thereby increasing donor confidence, which is an important factor in financial sustainability of the disaster and relief programs. However, it’s the entire team of the program managers, healthcare providers and policy makers who work together for effective pharmaceutical management by ensuring quality and availability of medicines and laboratory supplies as well as monitoring of patient adherence, patient safety, and drug resistance. The latter part is needed to rationalize the medicine use process during relief operations.

Evolution of LSS/SUMA to PIMS is a successful attempt to respond to the above stated challenges. The aim of this article is to share the experience of developing a service-driven-software for pharmaceutical supplies during emergency conditions and disasters, and the advantages gained.

## Development of the New Software

### Overview and Limitations of the Old Softwares in Use by WHO

In 1992, Humanitarian Supply Management System (SUMA) developed by PAN American Health Organization was first introduced in Latin America and the Caribbean to manage the medical donations to the disaster areas (SUMA Project-PAN American Health Organization, 2018). Later in August 2005, SUMA was upgraded to an interagency Logistic Support System (LSS) (Relief, 2005) which was designed to operate at national emergency management headquarters. The major functions were to establish the criteria to be used by the warehouse, consolidate the information, respond to queries, prepare reports to support the decision- making processes and promote inter-institutional coordination (UN Office for the Coordination of Humanitarian Affairs [OCHA], 2006). LSS is not a tracking system, but it provides an overview to the United Nation (UN) agencies, national authorities and other bodies about what has been pledged, in-pipeline, purchased, and donated. The key applications of LSS are to sort and label the incoming supplies, to classify the supplies into categories (e.g., pharmaceutical, non-pharmaceutical) and subcategories (e.g., analgesics, anti-infectives in pharmaceutical category), to handle the queries about the available items and provide reports, and to ensure the proof of delivery to the recipients (UN Office for the Coordination of Humanitarian Affairs [OCHA], 2006). The software was later expanded to include all types of donations including medicine, food and non-food items.

**FIGURE 1:**
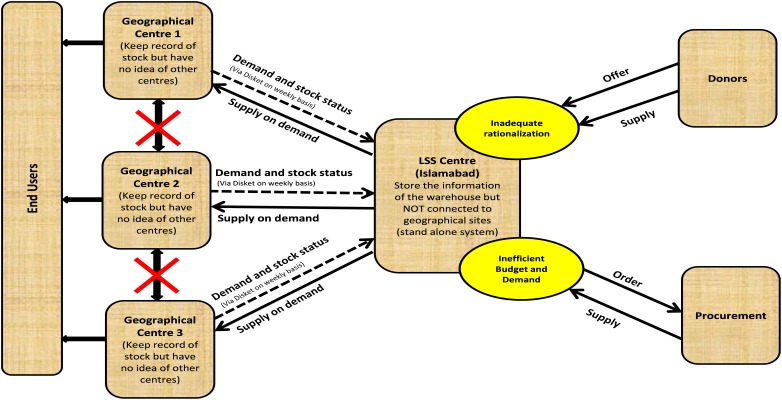
Operational scheme and deficiencies of LSS.

The LSS/SUMA was used in more than 33 major humanitarian disasters (PAH, 2018) around the world for tracking and reporting goods coming into a disaster area till 2008, however, the Pakistan earthquake in October 2005 was the first major disaster management operation handled using the LSS after its revision from SUMA in August 2005. Within 6 months after earthquake LSS was successfully deployed in the major affected districts of Pakistan including Mansehra, Bagh, Islamabad (WHO warehouse), Muzaffarabad, Balakot, Rawalakot, and Batagram to manage all the three phases of disasters (UN Office for the Coordination of Humanitarian Affairs [OCHA], 2006). A dedicated team of 6 pharmacists (which grew into a national team of 35 pharmacists in 5 years) were trained on LSS to meet the national needs in disaster management reaching the remotest affected areas. The stockpiles of essential medicines donation supplies with detailed information were inventoried on LSS database for managing the dispatch to the disaster-hit areas in the shortest possible time. All medical supply requests from field were entered through LSS request module which had an interface to pipeline module (goods in transit). All deliveries were managed through delivery module which can be used to create way bills (delivery challan/note). In this way, headquarter can coordinate the internal management of relief supplies, their distribution to facilities or field units and other organizations involved. An excel sheet, extracted from LSS, was used to setup LSS intranet work system. Information from each station was updated on weekly basis on this system. WHO Headquarter allowed access of the stock information enabling departments and field units to be aware of the categorized items and medicines in the warehouse and the balance available for distribution. Furthermore, the LSS improved information flow and thus increased transparency. Data import and export within and outside systems was advantageous. Thus, LSS served by sorting of donations, their inventory management and priority setting to improve access to disaster victims. The system has been widely supported by WHO and its use has been extended all over Pakistan not only in WHO warehouses but also at various locations of the Provincial Departments of Health. However, several deficiencies were also identified in LSS (Figure 1) during the relief operations in Pakistan. Some of these are detailed as follows:

#### Absence of Real-Time Reporting

Inability to cross link the central warehouse with other geographical sites was the major shortcoming experienced with the LSS, impeding the real time reporting. The LSS provincial WHO warehouses were not inter-linked with each other and to the WHO main warehouse at Islamabad, hereby hindering effective planning of contingencies and other timely actions. Disasters are accompanied by unpredictable and urgent need situations, which in case of medicines and pharmaceutical supplies can turn into a major life-threatening calamity involving masses and vast regions, spread of epidemics due to shortage of vaccines and antibiotics is one such example. Moreover, the needs arising in disaster situation are different from the routine pharmaceutical usage resulting in the extreme shortages of specific therapeutic agents and devices. Need of a cross-linked inventory network is inevitable to meet such situation in ideal sense. It was realized that the software, with the requirement of robustness and adaptability through some planning options, was available through pipeline module but that was ineffective. Cross linking through diskettes was also undertaken as an immediate measure to interlink the stand-alone sites but this made the software operations slow and vulnerable to viruses.

#### Lack of Customized Reporting

Reporting helps the donor through transparency and visibility of project performance and strengthens their trust. LSS was successful in generating different reports as required by donors in formats specific for mass communication perspective, but still certain graphs and customized reporting were missing. In-compatibility with other reporting systems (e.g., softwares used by MSF and some other major organizations) thus making it less adaptable. There was also a barrier in generating tailored reports needed time to time.

#### Absence of Popups

The tracking, tracing, and expiry popups to alert the users and decisions maker for appropriate actions were not present and hence limited the use of LSS. Popups and proactive inventory actions is of crucial importance in relief operations and emergency conditions due to less chances of availability of expert users in effected regions and less time available to carry out manual analysis of the inventory data and to design actions.

#### Absence of Procurement and Supply Quantification Tools

The basic procurement procedures including planning, forecasting, quantification, and purchase order generation were not covered adequately by LSS. Similarly, warehouse management tasks in LSS were limited to entries and delivery modules.

#### Limited Security, Access, and Audit Features

LSS is a multi-user software and user authorization is needed to authenticate and access the database securely. However, the LSS user permissions and authentications control is limited to only few features that includes restriction of users to login or view the certain allowed modules. Further the system lacks audit trail that records the transaction or actions performed by specific users. Moreover, the software was not suited for audit purposes because of unrestricted access of users to make changes in database, items names, categorizations of items and deletion of entries, etc.

#### Absence of Backup Features

LSS has built-in manual database backup and restoration features, however, it lacks auto backup. Automatic backups are needed to deal with the incidences of database failures and assume that a fault may cause data loss and so data archives are made as a safety measure. Alternate to this, making a second (or more) set of copies for data restoration from expected data losses is also used. A successful automatic backup system will be immune to situations such as fire, floods, and other natural disasters that could occur at the hard drives’ physical location. That is why automatic backup systems should be remote, and data restoration and retrieval should be located away from the original data storage location. Such remote automatic back up system was not available in LSS module.

### Customization of LSS and Key Features of the Future Application

The LSS team in Pakistan enabled health authorities and the WHO office to respond quickly and efficiently in the subsequent rescue and rehabilitation operation as needed by the country. Pakistan faced a series of natural and manmade disasters in the last decade (Ten Worst Disasters in Pakistan, 2011) effecting large number of people and covering vast geographical regions. These include floods in Sindh and Baluchistan-Cyclone Yemyin in 2007; the earthquake in Northeast of Awaran near Afghan border in the province of Baluchistan in 2008; the crisis due to Internally Displaced People (IDPs) of Swat and Waziristan in 2009 (UN Office for the Coordination of Humanitarian Affairs [OCHA], 2009) and heavy monsoon rains in coastal region of Sindh in 2011 affecting approx. 18 million people (Memon, 2012). Most of these disasters acquire a full-blown status in extremely short period of time like the 2011 crisis, where the crisis emerged just after 2 days of rain (Memon, 2012). The experiences of multiple and diverse nature of emergency needs in different regions and situations (Bukhari et al., 2010; Tordrup et al., 2013) motivated the supply chain managers to customize LSS into the final version of PIMS. The worst crisis in history of Pakistan was the “2010 Indus valley floods” affecting 20–23 million people at one time (IRIN, 2010; Ten Worst Disasters in Pakistan, 2011). This crisis was managed using the new software called PIMS.

Immediately after LSS deployment in Pakistan it went through several changes and modifications to better meet the needs of the local requirements. Table 1 includes the list of such upgradations that were included in PIMS. During this trial and error period the first intervention that was carried out included simplification and improving the categorization of items included in LSS/SUMA. In original version, there were multiple and complex categories and subcategories of medicines, consumables, food and other goods used in relief operations like tents. The step also aided in the revision of the inventory operations and product categories to fulfill the pharmaceutical supply chain requirements like consideration for temperature sensitive medicines or specialized handling of controlled substances.

**Table 1:** List of features absent in LSS and were introduced in PIMS.

	Exclusive features of PIMS
1.	Centralized data entry
2.	Transparency and real time reporting
3.	Tracking of end-to-end deliveries
4.	Customize therapeutic classification-based product entries
5.	Search for therapeutic alternates
6.	Customized stock baskets/kit assembly
7.	Shelfing and exact location entry in the premises
8.	Interactive user interface
9.	Network and enterprise configuration
10.	User authentications based on their roles
11.	Resource editor tool
12.	Expiry management and alert
13.	Supply chain management functions
14.	Customized reporting
15.	Advance graphs presentation
16.	Reliability of data


The second major customization step involved conversion of system from standalone/single client system to online system with consolidation of information on stocks from all locations enabling a functional real time reporting system. Another major operational intervention was the provision of multi-users authorization operable at various levels. Auto back-up and restoration was carried out to help speed-up the process without security lapse (unauthorized access). PIMS has a built-in program to take back up of database on daily basis as well as auto restoration in case of failure. This feature protects from loss of data and preservation of all the previously assigned user authorization and permissions.

In addition to the above features, the new software recorded the product data using the International Non-proprietary Names (INN) names in accordance with therapeutic classification of drugs and facilitated inclusion of brand names allowing search for therapeutic alternates and substitutes. The database contains about 2000 INNs of medicines along with strengths, dosage form, etc. In case of non-availability of the item entry, the software provides a portal for the entry of new pharmaceutical supply in accordance with the national or regional need.

A data entry fields for each product included strengths, dosage forms, routes of administration, batch numbers, expiry dates, delivery, sending and receiving dates, unit cost as well as temporary storage and location for quick access. The importance of sending date is of special concern for temperature sensitive medicines as well as in calculating the transportation time between various sites. Precautionary notes are added in software for temperature-, moisture- and light-sensitive medicines as well as for controlled and hazardous substances like narcotics for ensuring secure storage.

The software helps to prepare the expiry calendars for checking the levels of shelf life at any stage. The software generates expiry alerts and operates on FEFO (first expiry first out) principle. In case the near to expiry items cannot be used in the facility within that duration, the report is shared with main warehouse, for distribution to regions where the consumption of medicine is well-anticipated or demanded.

**FIGURE 2:**
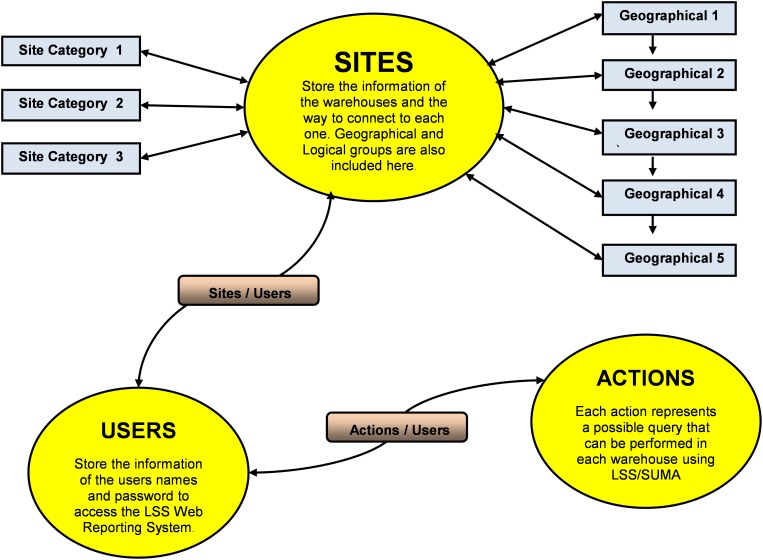
Operational scheme of PIMS.

The unique feature that makes the PIMS a service driven software for disaster management is the provisions to define stock basket and specialized medicine kits, a concept that links the epidemiological data with the predefined treatment protocols for use in selection of drugs to the disaster-stricken area. The operational scheme of PIMS is shown in Figure 2. PIMS was instrumental in helping the pharmacists in central warehouse and peripheral warehouse to customize kits using the information on product categories and on therapeutic alternates. The kits played a crucial role in efficient delivery of the stocks in need.

### Implementation

World Health Organization hired a team of pharmacists to ensure medicine quality, safety, and efficacy during supply chain operations in disaster areas. Since the onset of the crisis, WHO Pharmacists were engaged in sorting, procuring, distributing, and monitoring medicines to the displaced population. This team also provided training on use of PIMS for generating evidence based demands and monitoring rational use of medicines by comparing product consumptions against treatment protocol, and implementing procurement/contingency plans.

During the disaster relief operations PIMS was not only used by WHO rather more than 24 international and local healthcare NGOs also utilized it for managing and ordering medical supplies. PIMS success in disaster relief operations encouraged its use by the public sector facilities in diverse settings even after disaster phase was over. PIMS was installed at various public facilities including Executive District Officer Health (EDOH) and People Primary Health Care Initiative (PPHI) offices. Capacity building was also carried out for the other health care partners involved in the medicines supply chains during the relief operations. The donors and charity organizations who were trained on PIMS are enlisted in Supplementary Table 1.

Two editions of PIMS are available. PIMS central edition this is web-based and is mainly used by district/sub-regional supervisors to create/authorize/approve deliveries to the central level. Hospitals can use it as well for report and requisition. PIMS facility edition is for high volume facilities especially those considered to be first level referral hospitals or similar as well as regional hospitals and teaching hospitals. These editions operate by using a local area network (LAN) for stock issues from the stores and dispensing of drugs. PIMS Pakistan team assisted to rectify the data duplication error in pilot version and also provided recommendations for upgradation.

Pharmaceutical Information Management System also covers the financial aspects of the stocks. The software gives provision of entering unit price against each items thus informing the cost of each delivery and stock transfer. PIMS also supports in budget planning which is crucial for developing contingency plans. PIMS can collect the data from central hospitals, provincial hospitals, district hospitals, health centers with estimated values for modeling copayment schemes. Additional costs can be added thereby providing invoice with each transaction. This shows that the software is adaptable to diverse settings.

### Impact

The new system identifies gaps in supply chain of essential health commodities and is able to provide targeted support. For instance, PIMS monitored inventory of health products from the level of WHO warehouses up to provincial medical warehouses and its impact is seen by the visibility of the countrywide available stocks at any site and point in time within a couple of second. The system operates paper-less requiring minimum logistics and costs involved.

The re-categorization dramatically reduced the complexity of the tool and removed the duplication errors. It allowed the operator to work faster and with more convenience. Professional input of pharmacist was crucial for correct categorization and therapeutic classification of the pharmaceutical supplies. The pharmacist was thus realized as a crucial person needed for the accurate data structuring of the tool.

Use of the updated information on drug consumption data played crucial role for effective quantification and ordering throughout the supply chain. This consumption data was used to verify the disease morbidity data and vice versa. The use of new software avoided expiry of medicines to maximum and ensured transfer of surplus medicines to the region of need preventing stock overloads and stock outs, respectively.

The advancement in software from SUMA to LSS and then finally to PIMS improved the transparency in the management of humanitarian supplies and provided reports for sharing with donors, authorities, humanitarian agencies and the media in more effective and efficient manner thereby improving donor confidence and better logistic management.

## Review of the Findings

The PIMS operates on principles of pharmaceutical supply chain management model with four major components, namely product selection, quantification and procurement, inventory management, and serving patients. PIMS provides information to each of the component to make evidence-base decisions and manage the supply chain. Implementation of PIMS had two major positive effects firstly the data is used to reduce stock-outs and secondly it helped to carry out more informed decisions.

With the use of new software potential stock-outs were reduced by more than 85% at all WHO stores, while under-stock of the same commodities was also reduced by 60% at both levels. Any kind of stock-out (stock-out, potential stock-out, and under-stock) for life saving injectable has reduced significantly in 2013 in comparison with 2006. A figure of wastages of 1.3% was reported with the use of this advanced version of logistic software, which is lower than what was incurred in the major global disasters of last decade (Bukhari et al., 2010). The major reasons for the high wastages included short shelf life as well as absence of expiry data in the inventory management module of LSS (Bukhari et al., 2010).

The availability of logistics data has improved decision making at several levels of the system. A rational approach to procurement is achieved using PIMS, which not only provides the necessary data for selection of products required for next cycle of procurement, aid in budget estimation, but also keep in account the pipeline supplies to avoid over or under stocking. Pipeline stocks and delivered stocks for a location are accessible in the central warehouse with details up to batch, serialization and quantities. Availability of real time data at WHO central warehouse helped to carry out more accurate forecasting of pharmaceutical supplies needed in different field situations. This allowed in-depth, interactive discussion among partners to prepare, review and update the national needs for essential medicines to revise forecasting, fund-gap analysis, and supply planning. Availability of accurate data also adds transparency and confidence in the negotiation process, developing a more harmonized approach among stake holders.

Pharmaceutical Information Management System avoided misinformation in the stock details by the use of uniform policy for categorization and product names. This ruled out the possibility of unattended/dead stocks by generation of uniform reporting from all sites. PIMS database infrastructure enables transparency in the system as no entry could be deleted intentionally or by mistake. The application was helpful in the successful third-party audits as well as in gaining donor confidence and credibility of the humanitarian effort.

Based on the initial supply data in emergency relief process per capita consumption of essential medicines and health facilities utilization rates were calculated and used to design customized medicines kit which was incorporated in the next module that is PIMS. Table 2 shows customized health kits along with their coverage. Use of medicine packages by linking epidemiological data with drug utilization data to tackle disease outbreaks played crucial role in efficient decision making and planning of rescue and relief efforts.

**Table 2:** Customized health kits and their coverage for use in management of crisis situations.

Kit/package name	Coverage
Interagency emergency health kit (IEHK)	30,000 population for 1 month
Emergency health kit (EHK)	9,000 population for 1 month
Diarrheal disease kit (DDK)	500 severe to 1000 moderate interventions
Diarrheal treatment center kit (DTC)	Same coverage as of DDK (medicine + surgical + equipment)
Secondary healthcare kit	200,000 population for 3 months


Capacity to handle unreliable or unpredictable supplies during the disasters was increased as based on PIMS data many donation offers were rejected. With PIMS the primary components of the inventory that is the available and pipeline stocks, the consumption trends and the institutional or field requests all were viewed simultaneously giving a most reliable picture for the need of evaluation and assisting evidence-based decision making on the acceptance of donations. Unsolicited donations are identified as one of the reasons leading to high wastage and inventory holding costs in disasters. Consumption trends extracted from PIMS data helps in revising the customized kits according to seasonal variation in consumption. Precedence data from the previous year/season also helped in contingency planning and prepositioning of buffer/emergency stocks at vulnerable sites.

Emergence of unpredictable pharmaceutical needs of urgent nature, in the amounts unlikely to be used in routine settings is one of the major challenges experienced during a disaster (Tayfur and Benjamin, 2007). During the massive 2010 floods, need was identified for Primaquine which was not available in Pakistan but urgently required for control malaria outbreak in Southern part of Punjab province. The WHO Essential Medicines team traced large volume of donation stock with the help of centralized data base from another province. With the earlier version of LSS it was impossible to identify stocks so fast and effectively. Keeping this experience in view, recently the same approach is being adopted by computerization of inventories in 8 districts of Punjab in Pakistan.

The data and experience from the emergency operations in Pakistan were later helpful in supply chain management for medicinal product during humanitarian crisis in Haiti. The development of the best practices in medicine management during emergency operations was also supported by the data and reports accessible from PIMS.

Over the times PIMS has proven to be a user-friendly and efficient system which is adopted in Pakistan as well as in other countries including Sierra Leone, Afghanistan, Nepal, and Philippines with the help of the experts that were involved in the upgradation of LSS to PIMS.

## Overall Discussion

Use of centralized database linked with peripheral or satellite units is important component needed to design an efficient and cost contained system. The achievement of rational quantification, procurement and distribution of stock is not possible without centralized database. PIMS communicate stock needs quickly, efficiently, with a high level of confirmation and status follow-up. Further PIMS allows group of users to have access to the same information, allowing each group to use and analyze the data. This permits a proactive and a multi-pronged approach to supply-chain management, with teams in distribution hubs and procurement staff based in Islamabad working proactively to prevent product stock-outs.

Delivery of goods to disaster affected area mostly need specialized logistic arrangement for transportation and special permission to access war and conflict zones hence precision in planning and assurance of delivery of an evidence-based package of supplies is crucial to these life-saving efforts.

Pharmaceutical Information Management System provided institutional memory for each supplier assisted in the procurement of supplies, based on their performances calculated by criteria including the actual lead time. This developed a build in quality assurance system.

Non-availability of precedence data for defining pharmaceutical needs during disasters is an impediment to the anticipation of supply requests in the new crisis situations (Tayfur and Benjamin, 2007). Availability of a comprehensive logistic that is applicable to a large region and population provide a reliable data for safe forecasting and procurement planning, an important component of inventory management cycle.

## Conclusion

The use of PIMS has established an advanced role of information technology in the supply chain management that can help to save major losses of lives and to prevent aggravation of human suffering by ensuring fast and in time accessibility of required stocks at the needed place. The visibility of stocks in connected stations covered under PIMS provides cost effective supply enabling the right of patient to access medicine. The proactive nature of the software application helps in reducing undesired losses due to expiry and beforehand information on the storage and handling of supplies. It is foreseen that the application will not be limited to the use in humanitarian crisis but also be found equally effective in tackling global medicine shortage, access to orphan medicine, establishment of public medical services in resource limited settings due its built-in design of transparency, cost containment in procurement and distribution of medicines.

## Author Contributions

HR and KB conceived and designed the study. HR drafted the outline and wrote the initial draft of the Section “Discussion”. MU wrote the initial draft of the Section “Introduction”. MB wrote the initial draft of the Section “Overview of LSS”. WA wrote the initial draft of the Sections “Customization of LSS and Key Features of the Future Application” and “Review of the Findings”. AZ wrote the initial draft of the Sections “Implementation” and “Impact”, and MU wrote the initial draft of their respective sections. HR, WA, AZ, and MU refined the manuscript draft. HR, MU, and KB finalized the manuscript. All authors contributed to the manuscript revision and read and approved the submitted version.

## Conflict of Interest Statement

The authors declare that the research was conducted in the absence of any commercial or financial relationships that could be construed as a potential conflict of interest. The handling Editor and reviewer SH declared their involvement as co-editors in the Research Topic, and confirm the absence of any other collaboration.
