# Supplementary material for: A Shift From Logistic Software to Service Model: A Case Study of New Service-Driven-Software for Management of Emergency Supplies During Disasters and Emergency Conditions by WHO
Source: Front Pharmacol. 2019 May 7;10:473. doi: 10.3389/fphar.2019.00473 (PMC6514185; doi:10.3389/fphar.2019.00473)
Supplement: Supplementary file 1 [file Table_1.DOCX]

Table 1 S List of donor agencies trained for use PIMS for medicine supply management

| **S No.** | **Organization Name** | | **Province** | | **District** |  |
| --- | --- | --- | --- | --- | --- | --- |
| 1 | American Refugee Committee (ARC) | | Khyber Pakhtun Khwah | | Swat |  |
|  |  |  | Balocistan | | Sibi |  |
|  |  |  |  |  | Bolan |  |
|  |  |  |  |  | Quetta |  |
| 2 | Concern | | Khyber Pakhtun Khwah | | Charsadda |  |
| 3 | Community World Service Pakistan/Afghanistan  (CWS-P/A) | | Khyber Pakhtun Khwah | | Manshera |  |
|  |  |  |  |  | Kohistan |  |
|  |  |  |  |  | Swat |  |
| 4 | Helping hand for relief & development (HHRD) | | Khyber Pakhtun Khwah | | Buner |  |
|  |  |  |  |  | Swat |  |
|  |  |  |  |  | Nowshera |  |
|  |  |  |  |  | Charsadda |  |
|  |  |  |  |  | Lower Dir |  |
|  |  |  | Punjab | | Mianwali |  |
|  |  |  |  |  | D.G.Khan |  |
|  |  |  |  |  | Layyah |  |
|  |  |  |  |  | Muzaffargarh |  |
|  |  |  | Sindh | | Kashmore |  |
|  |  |  |  |  | Sukkur |  |
|  |  |  |  |  | Thatta |  |
| 5 | Human Appeal Int. | | Khyber Pakhtun Khwah | | Nowshera |  |
|  |  |  |  |  | Charsadda |  |
|  |  |  |  |  | Malakand |  |
| 6 | International Medical Corps(IMC) | | Khyber Pakhtun Khwah | | Hangu |  |
|  |  |  |  |  | Kohat |  |
|  |  |  |  |  | Charsadda |  |
|  |  |  |  |  | Peshawar |  |
|  |  |  |  |  | Nowshera |  |
|  |  |  |  |  | Swat |  |
|  |  |  |  |  | Buner |  |
|  |  |  | Punjab | | Muzaffargarh |  |
|  |  |  |  |  | Multan |  |
|  |  |  |  |  | Layyah |  |
|  |  |  |  |  | Rajanpur |  |
|  |  |  | Sindh | | Larkana |  |
|  |  |  |  |  | Shikarpur |  |
|  |  |  |  |  | Thatta |  |
|  |  |  |  |  | Qambar Shahdadkot |  |
| 7 | | Internatioanl Organization for Migration(IOM) | | Punjab | Muzaffargarh | |
|  |  |  |  |  | Rajanpur | |
| 8 | | Islamic Relief | | Khyber Pakhtun Khwah | Nowshera | |
|  |  |  |  |  | Charsadda | |
|  |  |  |  | Punjab | Muzaffargarh | |
| 9 | | Medecins du Monde France(MDM-F) | | Khyber Pakhtun Khwah | Buner | |
|  |  |  |  |  | Kohat | |
|  |  |  |  |  | Swabi | |
|  |  |  |  |  | Charsadda | |
|  |  |  |  |  | Nowshera | |
| 10 | | Merlin | | Khyber Pakhtun Khwah | Buner | |
|  |  |  |  |  | Nowshera | |
|  |  |  |  |  | Swat | |
|  |  |  |  |  | Charsadda | |
|  |  |  |  | Punjab | Muzaffargarh | |
| 11 | | Muslim Aid | | Khyber Pakhtun Khwah | Charsadda | |
|  |  |  |  |  | Nowshera | |
|  |  |  |  | Sindh | Thatta | |
|  |  |  |  |  | Qambar Shahdadkot | |
|  |  |  |  | Punjab | Rajanpur | |
| 12 | | National Rural Support Programe(NRSP) | | Khyber Pakhtun Khwah | D.G.Khan | |
|  |  |  |  |  | Charsadda | |
|  |  |  |  |  | Nowshera | |
|  |  |  |  | Punjab | Rajanpur | |
|  |  |  |  |  | D.G.Khan | |
|  |  |  |  |  | Bhakkar | |
|  |  |  |  |  | Mianwali | |
|  |  |  |  | Sindh | Thatta | |
|  |  |  |  |  | Badin | |
| 13 | | PAIMAN | | Khyber Pakhtun Khwah | Charsadda | |
|  |  |  |  |  | D.I.Khan | |
|  |  |  |  |  | Swat | |
|  |  |  |  | Sindh | Dadu | |
|  |  |  |  |  | Sukkur | |
|  |  |  |  |  | Khairpur | |
|  |  |  |  | Punjab | D.G.Khan | |
|  |  |  |  | Balocistan | Jaffarabad | |
| 14 | | Plan Pakistan | | Punjab | Layyah | |
|  |  |  |  |  |  |  |
|  |  |  |  |  | Rajanpur | |
| 15 | | Save the Children | | Khyber Pakhtun Khwah | Kohat | |
|  |  |  |  |  | Swat | |
|  |  |  |  |  | Buner | |
|  |  |  |  |  | D.I.Khan | |
|  |  |  |  | Sindh | Sukkur | |
|  |  |  |  |  | Shikarpur | |
|  |  |  |  | Punjab | Jacobabad | |
|  |  |  |  |  | Rajanpur | |
|  |  |  |  |  |  |  |
|  |  |  |  | Balochistan | Jaffarabad | |
|  |  |  |  |  | Jhal Magsi | |
| 16 | | SPC | | Khyber Pakhtun Khwah | Swat | |
| 17 | | The Johanniter International Assistance | | Khyber Pakhtun Khwah | Peshawar | |
|  |  |  |  |  | Kohat | |
| 18 | | World Vision | | Khyber Pakhtun Khwah | Lower Dir | |
|  |  |  |  |  |  |  |
|  |  |  |  |  |  |  |
|  |  |  |  | Sindh | Khairpur | |
| 19 | | Malteser International | | Khyber Pakhtun Khwah | Swat | |
|  |  |  |  |  | Kohistan | |
|  |  |  |  | Punjab | Rahim Yar Khan | |
| 20 | | CARE | | K.P.K | Swat | |
|  |  |  |  |  | Nowshera | |
|  |  |  |  |  | Charsadda | |
|  |  |  |  | Punjab | Muzaffargarh | |
|  |  |  |  |  | Rajanpur | |
|  |  |  |  | Sindh | Shikarpur | |
|  |  |  |  |  | Sukkur | |
|  |  |  |  |  | Qambar Shahdadkot | |
|  |  |  |  |  | Kashmore | |
| 21 | | National Rural Support Program | | Punjab | Rajanpur | |
|  |  |  |  |  | D.G.Khan | |
|  |  |  |  |  | Bhakkar | |
|  |  |  |  |  | Mianwali | |
| 22 | | International Organization for Migrants | | Punjab | Muzaffargarh | |
|  |  |  |  |  | Rajanpur | |
| 23 | | Helping Hand Relief and Development | | Punjab | Mianwali | |
|  |  |  |  |  | D.G.Khan | |
|  |  |  |  |  | Layyah | |
| 24 | | International Medical Corps | | Punjab | Muzaffargarh | |
|  |  |  |  |  | Layyah | |
|  |  |  |  |  | Multan | |
